# Supplementary material for: Polarization of beliefs as a consequence of the COVID-19 pandemic: The case of Spain
Source: PLoS One. 2021 Jul 13;16(7):e0254511. doi: 10.1371/journal.pone.0254511 (PMC8277027; doi:10.1371/journal.pone.0254511)
Supplement: S1 Text — Full explanation of the iterative proportional fitting procedure to weight data. Supporting analyses on “Personal beliefs, political preference and sociodemographic groups”, and “Effect of the pandemic on personal beliefs”. Additional reference included in Supplementary Methods. (DOCX) [file pone.0254511.s001.docx]

**Polarization of beliefs as a consequence of the COVID-19 pandemic: the case of Spain**

Short title: *Belief polarization after COVID-19 pandemic*

Javier Bernacer^1^*, Javier García-Manglano^2^, Eduardo Camina^1,3^ and Francisco Güell^1^

^1^Mind-Brain Group, Institute for Culture and Society (ICS)

University of Navarra. Pamplona, Spain

^2^Youth in Transition Group, Institute for Culture and Society (ICS), University of Navarra. Pamplona, Spain

^3^Faculty of Education and Psychology, University of Navarra. Pamplona, Spain

Supplementary Methods

Iterative proportional fitting

In order to correct the unbalance in sex, age and political preference of our sample with respect to the nation totals, analyses were also replicated in a weighted database. In detail, each single respondent was assigned a weight to correct over or underrepresentation of sociodemographic variables. We used iterative proportional fitting (i.e. raking) for this purpose, by means of ‘ipfraking’ tool in Stata [1]. There were two control variables: 1) a combination of sex and age group, 2) political preference. The reference values were obtained from the latest national census (July, 2020: Instituto Nacional de Estadística, ine.es), and from the results of the latest general elections (November 10, 2020: <https://resultados.elpais.com/elecciones/2019/generales/congreso/index.html>). They were as follows: female, total=18499217: 18-30 yr: 3134088; 31-40: 3127207; 41-50: 3887523; 51-60: 3522104; 60-80: 4828295. Male, total=17996500: 18-30: 3265150; 31-40: 3106166; 41-50: 3945858; 51-60: 3445536; 60-80: 4233790. Hence, the total population (18-80 yr) was 36495717. With respect to politics, in the latest elections 9911563 people voted left-sided parties, 8704019 voted right-sided parties, and 5892133 voted ‘other’ (centrist or nationalist) parties. These numbers were scaled up to add up the total population (18-80 yr). In conclusion, the control values for politics were 14759826 left-sided voters, 12961609 right-sided voters, 8774282 ‘other’ voters. Weight calculation was carried out independently for each wave, in order to correct for differences in sample sizes. Finally, weights were divided by 3 to achieve the final target population. In conclusion, this procedure assigns a weight to each participant in order to correct their under or overrepresentation in the sample. Analyses on the weighted database are performed in Stata, in general terms, with prefix ‘svy:’, after establishing the survey parameters with ‘svyset’ (see S1 Statistical reports).

Supplementary Results

Personal beliefs, political preference and sociodemographic groups

As an initial description of the data, we present the proportion of participants that strongly agreed (1), agreed (2), were neutral (3), disagreed (4) or strongly disagreed (5) with each item. These results include all participants (whole sample across waves, N=1706), and are summarized in S1 Table.

In short, the following propositions were supported (answered 1 or 2) by a majority (>50%) of participants: government authorities tend to be intrusive (item 4), all human beings deserve respect (item 7), weak people should be helped (item 9), being controlled by others is intolerable (item 10), most people have good intentions (item 11), and it is okay to use animals for medical research (item 12). On the other hand, a majority of participants were against the following propositions: there is nothing beyond death (item 2), the world is about to end (item 3), scientific progress can make humans immortal (item 5), and individual rights are more important than group necessities (item 6). Therefore, responses were mixed for items 1 (any failure can lead to a catastrophe) and 8 (God answers people’s prayers).

The average disagreement level for each item and wave is displayed in S1 Fig. This value is computed, for each item, as the sum of each disagreement level (1 to 5) multiplied by the proportion of participants that responded that disagreement level. For example, for item 1 and before COVID-19, 12% responded 1, 17% responded 2, 19% responded 3, 19% responded 4, 33% responded 5:

0.12*1 + 0.17*2 + 0.19*3 + 0.19*4 + 0.33*5 = 3.46.

We also analyzed the different endorsement of these propositions depending on political preference, sex, age and civil status, across all time points, that is, irrespective to the pandemic. To answer this, we ran an ordinal logistic regression for each item, in order to assess whether individual responses could be predicted by politics (0=right-sided voters; 1=left-sided voters; 2=other), sex (0=male, 1=female), age (0=18-30 yr, 1=31-40 yr, 2=41-50 yr, 3=51-60 yr, 4=60+ yr), civil status (0=single, 1=divorced, 2=widowed, 3=domestic partner, 4=married), COVID-19 sick relative (0=no, 1=yes) and COVID-19 deceased relative (0=no, 1=yes); thus, the effect of each independent variable was controlled for the effects of the remaining covariates. Note that in this case we did not include ‘wave’ in the model. Statistical results are shown in S2 Table.

Specifically on politics, all items except for 1, 3, 9 and 10 showed significant differences. In particular, right-sided voters disagreed more firmly (with respect to left-sided and ambiguous voters) with the idea of there being nothing beyond death (item 2), and with the capacity of science to achieve immortality (item 5). On the other hand, they agreed more firmly than the other political groups with the proposition of all human beings deserving respect (item 7), God answering people’s prayers (item 8), most people having good intentions (item 11), and animal experimentation being okay (item 12). Finally, left-sided voters differentially disagreed (with respect to right-sided and ambiguous voters) with authorities being intrusive (item 4), and the preeminence of individual rights over group necessities (item 6).

We also asked whether personal beliefs were different when grouping by sociodemographic variables: sex, age group and civil status. With respect to sex, using the same ordinal logistic regressions, female agreed more firmly than male participants with the idea of all human beings deserving respect (item 7), God answering people’s prayers (item 8), the necessity of helping those who cannot help themselves (item 9), and the intolerability of being controlled by others (item 10). On the other hand, women significantly disagreed with there being nothing beyond death (item 2), the capacity of science to achieve immortality (item 5), the preeminence of individual rights over group necessities (item 6) and animal experimentation being okay (item 12).

Regarding age, significant results were found for item 2 (“There is nothing beyond death”), item 5 (“Scientific progress can help us overcome death”), item 6 (“Individual rights are more important than group necessities”), item 7 (“All human beings deserve respect”), item 8 (“God answers people’s prayers”), item 10 (“Being controlled by others is intolerable”), and item 11 (“Most people generally have good intentions”). The most remarkable effects were those of items 5, 8 and 11: whereas the tendency towards disagreement increased in older groups with respect to the capacity of science to overcome death, participants over 40 showed a stronger agreement (with respect to the 18-30 yr group) with God answering prayers and people’s good intentions.

Finally, with regards to civil status, significant results were found for items 1 (“Any failure can lead to a catastrophe”), item 4 (“Government authorities tend to be intrusive”), item 8 (“God answers people’s prayers”), item 11 (“Most people generally have good intentions”) and item 12 (“It is ok to use animals for medical research”). In all cases, married respondents agreed more strongly with these propositions compared with single participants.

Effect of the pandemic on personal beliefs

In the main text, we present the change in the endorsement of the 12 propositions as a consequence of the COVID-19 pandemic. In order to further explore these differences, for every item, we statistically compared the percentage of participants that responded each disagreement level (1 to 5) at each wave (see S3 Table). Thus, chi-squared tests were performed for each item of the survey using the ‘tabulate’ command in Stata. The null hypothesis of this test is that the proportion of responses to each disagreement level is unchanged throughout the three waves. Since this chi-squared test provides a single significance value (for each item of the survey), we analyzed the adjusted residuals with the ‘tabchi’ command in order to detect the main contributors to the significant results. This command provides observed and expected frequencies, as well as adjusted residuals.

Data are summarized in S3 Table, and the results of both commands (‘tabulate’ and ‘tabchi’) are included in S1 Statistical reports. We present here the main contributors to each significant overall result. With respect to item 1, the pandemic largely decreased the proportion of participants that strongly disagreed (answering 5) with ‘any failure can lead to a catastrophe’ (before, 33.3%; outbreak, 5.7%; de-escalation, 3.4%). Respondents were against the idea of there being nothing beyond death (item 2) mainly before the pandemic, and this attitude was restored during the de-escalation (answering 4 or 5: before, 64.6%; outbreak, 51.8%; de-escalation: 62%). Interestingly, the effect of the pandemic outbreak on items 3 (an imminent end of the world) and 4 (intrusive government authorities) were contrary to expectations: the proportion of participants that disagreed (answering 4 or 5) with these propositions increased after the outbreak (item 3: before, 71%, outbreak, 84.2%; de-escalation, 79.6%; item 4: before, 12.8%, outbreak, 30.7%; de-escalation, 22%). With regards to item 6, the declaration of the state of alarm was associated with a disagreement (answering 4 or 5) on the preeminence of individual rights over group necessities, although this effect faded away in the de-escalation (before, 49.6%; outbreak, 64.9%; de-escalation, 53.29%). The firmness of believing in God’s answering people’s prayers fluctuated between time points: it decreased in the outbreak (before, 46.5% answered 1; outbreak, 33.6%) but increased again in the de-escalation (46.5%). Strong disagreement (answering 5) with this item also fluctuated: before, 16.7%; outbreak, 22.4%; de-escalation, 12.2%). The outbreak, which entailed the lockdown of the whole country, was associated with a weaker agreement on item 10: “being controlled by others is intolerable” (before, 70.2% of participants answered 1; outbreak, 49.8%; de-escalation, 56.5%). The pandemic attenuated the firmness in believing that people in general have good intentions (item 11: before, 52% scored 1; outbreak, 18.6%, de-escalation, 17.9%). Finally, the proportion of participants that strongly agreed with animal experimentation decreased after the outbreak (before: 45.5%; outbreak: 23.4%; de-escalation: 19.7), although responses transferred to a weaker agreement (answering 2), rather than turning into disagreement.

Supplementary Reference

1. Kolenikov S. Calibrating Survey Data using Iterative Proportional Fitting (Raking). Stata J. 2014;14: 22–59. doi:https://doi.org/10.1177/1536867X1401400104
