# Supplementary material for: Polarization of beliefs as a consequence of the COVID-19 pandemic: The case of Spain
Source: PLoS One. 2021 Jul 13;16(7):e0254511. doi: 10.1371/journal.pone.0254511 (PMC8277027; doi:10.1371/journal.pone.0254511)
Supplement: S1 Table — Concerning answers, 1 = “I agree, and I would continue to agree even if I were shown ‘irrefutable’ proof to the contrary”; 2 = “I agree, although I could change my mind if I were shown strong evidence”; 3 = “I neither agree nor disagree”; 4 = “I disagree, although I could change my mind if I were shown strong evidence”; 5 = “I disagree, and I would continue to disagree even if I were shown ‘irrefutable’ proof”. (DOCX) [file pone.0254511.s004.docx]

|  | Proposition | 1 | 2 | 3 | 4 | 5 |
| --- | --- | --- | --- | --- | --- | --- |
| 1 | I think that any failure can lead to a catastrophe | 7 | 34 | 19.5 | 32.1 | 7.4 |
| 2 | I think that there is nothing beyond death | 11.6 | 20.5 | 12.3 | 18.5 | 37.1 |
| 3 | I think that the world is about to end | 1.3 | 3.1 | 13.7 | 52.9 | 29 |
| 4 | I think that government authorities tend to be intrusive and controlling | 20.1 | 36 | 16.7 | 21.4 | 5.8 |
| 5 | I think that scientific progress can help us overcome death and live forever | 2.4 | 13.9 | 9 | 35.2 | 39.5 |
| 6 | I think that individual rights are more important than the needs of any group | 6.5 | 12.7 | 20 | 36.1 | 24.7 |
| 7 | I think that all human beings deserve respect | 75.2 | 16.5 | 1.8 | 2.5 | 4 |
| 8 | I think that God answers people’s prayers | 38.1 | 8.6 | 19 | 15.1 | 19.2 |
| 9 | I think that one should help those who are weak and cannot help themselves | 75.4 | 19.8 | 2.8 | 0.8 | 1.2 |
| 10 | I think that being controlled or dominated by others is intolerable | 53 | 25.3 | 10.8 | 7.9 | 2.9 |
| 11 | I think that most people generally have good intentions | 20.9 | 47.2 | 18.9 | 9.9 | 3.1 |
| 12 | I think that it is okay to use animals for medical research | 24.2 | 41.7 | 15.9 | 10.8 | 7.4 |
