# Supplementary material for: Polarization of beliefs as a consequence of the COVID-19 pandemic: The case of Spain
Source: PLoS One. 2021 Jul 13;16(7):e0254511. doi: 10.1371/journal.pone.0254511 (PMC8277027; doi:10.1371/journal.pone.0254511)
Supplement: S2 Table — Each model included item response (1 = strong agreement… 5 = strong disagreement) as dependent variable, political preference and wave as predictors, and sex, age, civil status, COVID-19 sick acquaintance and COVID-19 deceased relative as covariates. Note that positive values of z and OR greater than 1 indicate a stronger disagreement with the proposition. Number of observations = 1650. OR, odds ratio; SE, standard error. (DOCX) [file pone.0254511.s005.docx]

| Item 1 | Any failure can lead to a catastrophe | | | | | | |
| --- | --- | --- | --- | --- | --- | --- | --- |
|  | Model | LR χ^2^(15)=58.24, p<0.0001, pseudo-R^2^=0.0125 | | | | | |
|  |  |  | OR | SE | 95% CI | z | p |
|  |  | Left (vs Right) | 1.05 | 0.11 | 0.86,1.29 | 0.52 | 0.604 |
|  |  | Other (vs Right) | 0.95 | 0.12 | 0.73,1.22 | -0.43 | 0.671 |
|  |  | Female (vs Male) | 1.01 | 0.09 | 0.84,1.22 | 0.12 | 0.903 |
|  |  | 31-40 yr (vs 18-30) | 0.92 | 0.13 | 0.70,1.21 | -0.62 | 0.538 |
|  |  | 41-50 yr (vs 18-30) | 1.13 | 0.18 | 0.83,1.53 | 0.77 | 0.442 |
|  |  | 51-60 yr (vs 18-30) | 0.95 | 0.16 | 0.67,1.33 | -0.31 | 0.755 |
|  |  | 60+ yr (vs 18-30) | 1.02 | 0.20 | 0.69,1.50 | 0.10 | 0.917 |
|  |  | Divorced (vs single) | 0.78 | 0.22 | 0.44,1.36 | -0.87 | 0.383 |
|  |  | Widowed (vs single) | 1.09 | 0.45 | 0.49,2.43 | 0.22 | 0.823 |
|  |  | D. partner (vs single) | 0.61 | 0.13 | 0.40,0.94 | -2.23 | 0.026 |
|  |  | Married (vs single) | 0.76 | 0.10 | 0.59,0.97 | -2.17 | 0.030 |
|  |  | Sick relative (vs no) | 0.98 | 0.09 | 0.81,1.18 | -0.21 | 0.834 |
|  |  | Dead relative (vs no) | 0.55 | 0.17 | 0.30,1.02 | -1.89 | 0.059 |
|  |  |  |  |  |  |  |  |
| Item 2 | **There is nothing beyond death** | | | | | | |
|  | Model | LR χ^2^(15)=354.69, p<0.0001, pseudo-R^2^=0.0710 | | | | | |
|  |  |  | OR | SE | 95% CI | z | p |
|  |  | Left (vs Right) | 0.18 | 0.02 | 0.15,0.23 | -15.52 | <0.001 |
|  |  | Other (vs Right) | 0.36 | 0.05 | 0.28,0.46 | -7.85 | <0.001 |
|  |  | Female (vs Male) | 1.50 | 0.14 | 1.25,1.81 | 4.32 | <0.001 |
|  |  | 31-40 yr (vs 18-30) | 1.01 | 0.14 | 0.76,1.33 | 0.05 | 0.964 |
|  |  | 41-50 yr (vs 18-30) | 1.38 | 0.22 | 1.01,1.89 | 2.02 | 0.044 |
|  |  | 51-60 yr (vs 18-30) | 0.95 | 0.17 | 0.67,1.35 | -0.29 | 0.771 |
|  |  | 60+ yr (vs 18-30) | 1.15 | 0.23 | 0.77,1.71 | 0.70 | 0.484 |
|  |  | Divorced (vs single) | 0.80 | 0.24 | 0.44,1.45 | -0.73 | 0.465 |
|  |  | Widowed (vs single) | 0.95 | 0.39 | 0.42,2.12 | -0.14 | 0.892 |
|  |  | D. partner (vs single) | 0.75 | 0.16 | 0.48,1.15 | -1.32 | 0.187 |
|  |  | Married (vs single) | 1.13 | 0.15 | 0.87,1.46 | 0.92 | 0.358 |
|  |  | Sick relative (vs no) | 1.36 | 0.13 | 1.12,1.65 | 3.13 | 0.002 |
|  |  | Dead relative (vs no) | 1.14 | 0.38 | 0.59,2.20 | 0.39 | 0.697 |
|  |  |  |  |  |  |  |  |
| Item 3 | **The world is about to end** | | | | | | |
|  | Model | LR χ^2^(15)=28.70, p=0.0176, pseudo-R^2^=0.0077 | | | | | |
|  |  |  | OR | SE | 95% CI | z | p |
|  |  | Left (vs Right) | 0.98 | 0.11 | 0.79,1.21 | -0.20 | 0.840 |
|  |  | Other (vs Right) | 0.82 | 0.11 | 0.62,1.08 | -1.43 | 0.152 |
|  |  | Female (vs Male) | 0.96 | 0.09 | 0.79,1.16 | -0.46 | 0.648 |
|  |  | 31-40 yr (vs 18-30) | 0.93 | 0.14 | 0.69,1.25 | -0.48 | 0.629 |
|  |  | 41-50 yr (vs 18-30) | 1.12 | 0.19 | 0.81,1.55 | 0.68 | 0.494 |
|  |  | 51-60 yr (vs 18-30) | 1.03 | 0.19 | 0.71,1.48 | 0.15 | 0.884 |
|  |  | 60+ yr (vs 18-30) | 0.77 | 0.16 | 0.51,1.16 | -1.25 | 0.210 |
|  |  | Divorced (vs single) | 0.84 | 0.26 | 0.46,1.54 | -0.57 | 0.567 |
|  |  | Widowed (vs single) | 0.75 | 0.35 | 0.31,1.87 | -0.61 | 0.539 |
|  |  | D. partner (vs single) | 0.77 | 0.18 | 0.49,1.21 | -1.13 | 0.257 |
|  |  | Married (vs single) | 0.95 | 0.13 | 0.72,1.24 | -0.39 | 0.694 |
|  |  | Sick relative (vs no) | 1.16 | 0.12 | 0.95,1.42 | 1.45 | 0.148 |
|  |  | Dead relative (vs no) | 0.62 | 0.21 | 0.32,1.20 | -1.42 | 0.154 |
|  |  |  |  |  |  |  |  |
| Item 4 | **Government authorities tend to be intrusive and controlling** | | | | | | |
|  | Model | LR χ^2^(15)=86.72, p<0.0001, pseudo-R^2^=0.0177 | | | | | |
|  |  |  | OR | SE | 95% CI | z | p |
|  |  | Left (vs Right) | 1.56 | 0.16 | 1.28,1.91 | 4.33 | <0.001 |
|  |  | Other (vs Right) | 1.10 | 0.14 | 0.85,1.41 | 0.71 | 0.475 |
|  |  | Female (vs Male) | 1.10 | 0.10 | 0.92,1.32 | 1.07 | 0.286 |
|  |  | 31-40 yr (vs 18-30) | 0.86 | 0.12 | 0.65,1.13 | -1.10 | 0.271 |
|  |  | 41-50 yr (vs 18-30) | 0.87 | 0.13 | 0.64,1.17 | -0.93 | 0.353 |
|  |  | 51-60 yr (vs 18-30) | 0.72 | 0.12 | 0.51,1.01 | -1.90 | 0.057 |
|  |  | 60+ yr (vs 18-30) | 0.76 | 0.15 | 0.52,1.11 | -1.40 | 0.163 |
|  |  | Divorced (vs single) | 0.80 | 0.23 | 0.45,1.39 | -0.79 | 0.427 |
|  |  | Widowed (vs single) | 0.85 | 0.35 | 0.38,1.91 | -0.39 | 0.694 |
|  |  | D. partner (vs single) | 0.96 | 0.21 | 0.63,1.48 | -0.16 | 0.873 |
|  |  | Married (vs single) | 0.77 | 0.10 | 0.60,0.99 | -2.04 | 0.041 |
|  |  | Sick relative (vs no) | 0.82 | 0.08 | 0.68,0.98 | -2.12 | 0.034 |
|  |  | Dead relative (vs no) | 0.55 | 0.17 | 0.29,1.00 | -1.95 | 0.051 |
|  |  |  |  |  |  |  |  |
| Item 5 | **Scientific progress can help us overcome death and live forever** | | | | | | |
|  | Model | LR χ^2^(15)=133.89, p<0.0001, pseudo-R^2^=0.0310 | | | | | |
|  |  |  | OR | SE | 95% CI | z | p |
|  |  | Left (vs Right) | 0.49 | 0.05 | 0.40,0.61 | -6.71 | <0.001 |
|  |  | Other (vs Right) | 0.51 | 0.07 | 0.39,0.66 | -5.07 | <0.001 |
|  |  | Female (vs Male) | 1.37 | 0.13 | 1.13,1.64 | 3.27 | 0.001 |
|  |  | 31-40 yr (vs 18-30) | 1.23 | 0.18 | 0.92,1.63 | 1.42 | 0.156 |
|  |  | 41-50 yr (vs 18-30) | 1.50 | 0.24 | 1.09,2.06 | 2.51 | 0.012 |
|  |  | 51-60 yr (vs 18-30) | 1.41 | 0.25 | 1.00,2.00 | 1.95 | 0.051 |
|  |  | 60+ yr (vs 18-30) | 1.93 | 0.38 | 1.30,2.85 | 3.30 | 0.001 |
|  |  | Divorced (vs single) | 0.66 | 0.18 | 0.38,1.14 | -1.49 | 0.135 |
|  |  | Widowed (vs single) | 0.49 | 0.21 | 0.22,1.12 | -1.69 | 0.092 |
|  |  | D. partner (vs single) | 0.67 | 0.14 | 0.44,1.02 | -1.88 | 0.059 |
|  |  | Married (vs single) | 1.23 | 0.16 | 0.95,1.60 | 1.58 | 0.113 |
|  |  | Sick relative (vs no) | 1.37 | 0.14 | 1.13,1.67 | 3.18 | 0.001 |
|  |  | Dead relative (vs no) | 0.90 | 0.30 | 0.47,1.75 | -0.29 | 0.775 |
|  |  |  |  |  |  |  |  |
| Item 6 | **Individual rights are more important than the needs of any group** | | | | | | |
|  | Model | LR χ^2^(15)=70.23, p<0.0001, pseudo-R^2^=0.0144 | | | | | |
|  |  |  | OR | SE | 95% CI | z | p |
|  |  | Left (vs Right) | 1.46 | 0.15 | 1.19,1.78 | 3.66 | <0.001 |
|  |  | Other (vs Right) | 1.21 | 0.16 | 0.94,1.56 | 1.49 | 0.137 |
|  |  | Female (vs Male) | 1.48 | 0.14 | 1.23,1.77 | 4.21 | <0.001 |
|  |  | 31-40 yr (vs 18-30) | 1.26 | 0.18 | 0.96,1.66 | 1.66 | 0.097 |
|  |  | 41-50 yr (vs 18-30) | 1.17 | 0.18 | 0.87,1.58 | 1.03 | 0.304 |
|  |  | 51-60 yr (vs 18-30) | 1.47 | 0.25 | 1.05,2.05 | 2.26 | 0.024 |
|  |  | 60+ yr (vs 18-30) | 1.20 | 0.23 | 0.82,1.75 | 0.94 | 0.347 |
|  |  | Divorced (vs single) | 0.97 | 0.28 | 0.55,1.72 | -0.10 | 0.923 |
|  |  | Widowed (vs single) | 0.83 | 0.32 | 0.38,1.78 | -0.48 | 0.629 |
|  |  | D. partner (vs single) | 1.19 | 0.26 | 0.78,1.83 | 0.81 | 0.416 |
|  |  | Married (vs single) | 1.12 | 0.14 | 0.88,1.44 | 0.93 | 0.352 |
|  |  | Sick relative (vs no) | 1.09 | 0.11 | 0.90,1.32 | 0.93 | 0.353 |
|  |  | Dead relative (vs no) | 1.09 | 0.34 | 0.59,1.99 | 0.27 | 0.787 |
|  |  |  |  |  |  |  |  |
| Item 7 | **All human beings deserve respect** | | | | | | |
|  | Model | LR χ^2^(15)=67.33, p<0.0001, pseudo-R^2^=0.0255 | | | | | |
|  |  |  | OR | SE | 95% CI | z | p |
|  |  | Left (vs Right) | 1.41 | 0.18 | 1.09,1.82 | 2.63 | 0.008 |
|  |  | Other (vs Right) | 1.49 | 0.24 | 1.08,2.06 | 2.41 | 0.016 |
|  |  | Female (vs Male) | 0.50 | 0.06 | 0.40,0.64 | -5.77 | <0.001 |
|  |  | 31-40 yr (vs 18-30) | 1.79 | 0.32 | 1.27,2.53 | 3.31 | 0.001 |
|  |  | 41-50 yr (vs 18-30) | 1.27 | 0.25 | 0.86,1.87 | 1.20 | 0.230 |
|  |  | 51-60 yr (vs 18-30) | 1.18 | 0.26 | 0.76,1.83 | 0.75 | 0.453 |
|  |  | 60+ yr (vs 18-30) | 1.04 | 0.26 | 0.63,1.70 | 0.14 | 0.888 |
|  |  | Divorced (vs single) | 1.14 | 0.42 | 0.56,2.33 | 0.37 | 0.714 |
|  |  | Widowed (vs single) | 1.42 | 0.72 | 0.53,3.84 | 0.69 | 0.487 |
|  |  | D. partner (vs single) | 1.19 | 0.32 | 0.70,2.00 | 0.64 | 0.523 |
|  |  | Married (vs single) | 0.90 | 0.15 | 0.65,1.24 | -0.64 | 0.519 |
|  |  | Sick relative (vs no) | 0.73 | 0.09 | 0.57,0.94 | -2.46 | 0.014 |
|  |  | Dead relative (vs no) | 1.04 | 0.42 | 0.47,2.31 | 0.10 | 0.921 |
|  |  |  |  |  |  |  |  |
| Item 8 | **God answers people’s prayers** | | | | | | |
|  | Model | LR χ^2^(15)=539.41, p<0.0001, pseudo-R^2^=0.1090 | | | | | |
|  |  |  | OR | SE | 95% CI | z | p |
|  |  | Left (vs Right) | 8.99 | 1.03 | 7.19,11.25 | 19.25 | <0.001 |
|  |  | Other (vs Right) | 3.17 | 0.42 | 2.44,4.12 | 8.65 | <0.001 |
|  |  | Female (vs Male) | 0.63 | 0.06 | 0.53,0.77 | -4.71 | <0.001 |
|  |  | 31-40 yr (vs 18-30) | 1.02 | 0.15 | 0.77,1.36 | 0.14 | 0.889 |
|  |  | 41-50 yr (vs 18-30) | 0.68 | 0.11 | 0.50,0.93 | -2.38 | 0.017 |
|  |  | 51-60 yr (vs 18-30) | 0.72 | 0.13 | 0.51,1.03 | -1.79 | 0.074 |
|  |  | 60+ yr (vs 18-30) | 0.50 | 0.10 | 0.33,0.76 | -3.30 | 0.001 |
|  |  | Divorced (vs single) | 0.81 | 0.23 | 0.46,1.43 | -0.72 | 0.472 |
|  |  | Widowed (vs single) | 1.63 | 0.69 | 0.71,3.75 | 1.16 | 0.245 |
|  |  | D. partner (vs single) | 1.41 | 0.32 | 0.91,2.19 | 1.53 | 0.126 |
|  |  | Married (vs single) | 0.78 | 0.10 | 0.60,1.02 | -1.83 | 0.068 |
|  |  | Sick relative (vs no) | 0.71 | 0.07 | 0.60,0.87 | -3.37 | 0.001 |
|  |  | Dead relative (vs no) | 0.93 | 0.32 | 0.47,1.82 | -0.21 | 0.832 |
|  |  |  |  |  |  |  |  |
| Item 9 | **One should help those who are weak and cannot help themselves** | | | | | | |
|  | Model | LR χ^2^(15)=21.38, p=0.1253, pseudo-R^2^=0.0090 | | | | | |
|  |  |  | OR | SE | 95% CI | z | p |
|  |  | Left (vs Right) | 1.12 | 0.15 | 0.86,1.44 | 0.86 | 0.391 |
|  |  | Other (vs Right) | 1.27 | 0.21 | 0.92,1.75 | 1.46 | 0.146 |
|  |  | Female (vs Male) | 0.79 | 0.09 | 0.62,0.99 | -2.02 | 0.043 |
|  |  | 31-40 yr (vs 18-30) | 1.01 | 0.19 | 0.71,1.46 | 0.10 | 0.920 |
|  |  | 41-50 yr (vs 18-30) | 1.11 | 0.22 | 0.75,1.63 | 0.52 | 0.602 |
|  |  | 51-60 yr (vs 18-30) | 0.95 | 0.21 | 0.61,1.47 | -0.25 | 0.803 |
|  |  | 60+ yr (vs 18-30) | 0.97 | 0.24 | 0.60,1.58 | -0.12 | 0.906 |
|  |  | Divorced (vs single) | 1.51 | 0.53 | 0.76,3.02 | 1.18 | 0.239 |
|  |  | Widowed (vs single) | 1.47 | 0.75 | 0.54,4.02 | 0.75 | 0.454 |
|  |  | D. partner (vs single) | 0.74 | 0.22 | 0.41,1.33 | -1.00 | 0.315 |
|  |  | Married (vs single) | 0.98 | 0.16 | 0.71,1.35 | -0.11 | 0.912 |
|  |  | Sick relative (vs no) | 0.78 | 0.09 | 0.61,0.99 | -1.97 | 0.049 |
|  |  | Dead relative (vs no) | 0.96 | 0.39 | 0.43,2.14 | -0.09 | 0.927 |
|  |  |  |  |  |  |  |  |
| Item 10 | **Being controlled or dominated by others is intolerable** | | | | | | |
|  | Model | LR χ^2^(15)=63.77, p<0.0001, pseudo-R^2^=0.0157 | | | | | |
|  |  |  | OR | SE | 95% CI | z | p |
|  |  | Left (vs Right) | 0.92 | 0.10 | 0.74,1.13 | -0.79 | 0.429 |
|  |  | Other (vs Right) | 1.07 | 0.15 | 0.82,1.41 | 0.52 | 0.602 |
|  |  | Female (vs Male) | 0.71 | 0.07 | 0.59,0.87 | -3.40 | 0.001 |
|  |  | 31-40 yr (vs 18-30) | 1.07 | 0.16 | 0.80,1.42 | 0.47 | 0.640 |
|  |  | 41-50 yr (vs 18-30) | 0.92 | 0.15 | 0.67,1.27 | -0.51 | 0.611 |
|  |  | 51-60 yr (vs 18-30) | 0.92 | 0.17 | 0.64,1.32 | -0.43 | 0.664 |
|  |  | 60+ yr (vs 18-30) | 0.46 | 0.10 | 0.30,0.72 | -3.46 | 0.001 |
|  |  | Divorced (vs single) | 1.12 | 0.36 | 0.60,2.10 | 0.38 | 0.703 |
|  |  | Widowed (vs single) | 0.33 | 0.19 | 0.11,1.03 | -1.91 | 0.056 |
|  |  | D. partner (vs single) | 0.95 | 0.23 | 0.60,1.52 | -0.20 | 0.842 |
|  |  | Married (vs single) | 0.88 | 0.12 | 0.67,1.15 | -0.95 | 0.343 |
|  |  | Sick relative (vs no) | 1.02 | 0.10 | 0.83,1.24 | 0.18 | 0.855 |
|  |  | Dead relative (vs no) | 0.77 | 0.27 | 0.39,1.54 | -0.72 | 0.470 |
|  |  |  |  |  |  |  |  |
| Item 11 | **Most people generally have good intentions** | | | | | | |
|  | Model | LR χ^2^(15)=153.99, p<0.0001, pseudo-R^2^=0.0350 | | | | | |
|  |  |  | OR | SE | 95% CI | z | p |
|  |  | Left (vs Right) | 1.22 | 0.13 | 0.99,1.50 | 1.87 | 0.061 |
|  |  | Other (vs Right) | 1.37 | 0.18 | 1.05,1.79 | 2.35 | 0.019 |
|  |  | Female (vs Male) | 1.02 | 0.10 | 0.84,1.23 | 0.18 | 0.854 |
|  |  | 31-40 yr (vs 18-30) | 0.99 | 0.14 | 0.75,1.32 | -0.04 | 0.968 |
|  |  | 41-50 yr (vs 18-30) | 0.66 | 0.11 | 0.48,0.90 | -2.61 | 0.009 |
|  |  | 51-60 yr (vs 18-30) | 0.44 | 0.08 | 0.31,0.62 | -4.61 | <0.001 |
|  |  | 60+ yr (vs 18-30) | 0.42 | 0.09 | 0.28,0.63 | -4.20 | <0.001 |
|  |  | Divorced (vs single) | 0.93 | 0.28 | 0.51,1.69 | -0.23 | 0.816 |
|  |  | Widowed (vs single) | 1.13 | 0.49 | 0.48,2.66 | 0.28 | 0.782 |
|  |  | D. partner (vs single) | 0.75 | 0.18 | 0.47,1.19 | -1.23 | 0.220 |
|  |  | Married (vs single) | 0.70 | 0.09 | 0.54,0.91 | -2.68 | 0.007 |
|  |  | Sick relative (vs no) | 0.85 | 0.08 | 0.70,1.03 | -1.64 | 0.100 |
|  |  | Dead relative (vs no) | 0.70 | 0.22 | 0.38,1.31 | -1.11 | 0.267 |
|  |  |  |  |  |  |  |  |
| Item 12 | **It is okay to use animals for medical research** | | | | | | |
|  | Model | LR χ^2^(15)=301.78, p<0.0001, pseudo-R^2^=0.0640 | | | | | |
|  |  |  | OR | SE | 95% CI | z | p |
|  |  | Left (vs Right) | 3.28 | 0.35 | 2.65,4.05 | 11.01 | <0.001 |
|  |  | Other (vs Right) | 2.64 | 0.35 | 2.03,3.43 | 7.22 | <0.001 |
|  |  | Female (vs Male) | 2.18 | 0.21 | 1.80,2.63 | 8.06 | <0.001 |
|  |  | 31-40 yr (vs 18-30) | 1.25 | 0.18 | 0.94,1.66 | 1.57 | 0.117 |
|  |  | 41-50 yr (vs 18-30) | 1.21 | 0.19 | 0.89,1.65 | 1.20 | 0.229 |
|  |  | 51-60 yr (vs 18-30) | 1.23 | 0.22 | 0.87,1.74 | 1.18 | 0.240 |
|  |  | 60+ yr (vs 18-30) | 0.73 | 0.14 | 0.49,1.08 | -1.59 | 0.113 |
|  |  | Divorced (vs single) | 1.47 | 0.44 | 0.82,2.63 | 1.29 | 0.198 |
|  |  | Widowed (vs single) | 0.92 | 0.37 | 0.41,2.02 | -0.22 | 0.826 |
|  |  | D. partner (vs single) | 1.72 | 0.39 | 1.11,2.67 | 2.43 | 0.015 |
|  |  | Married (vs single) | 0.72 | 0.09 | 0.56,0.93 | -2.50 | 0.012 |
|  |  | Sick relative (vs no) | 0.65 | 0.06 | 0.54,0.79 | -4.33 | <0.001 |
|  |  | Dead relative (vs no) | 1.24 | 0.38 | 0.67,2.28 | 0.68 | 0.494 |
