# Supplementary material for: Polarization of beliefs as a consequence of the COVID-19 pandemic: The case of Spain
Source: PLoS One. 2021 Jul 13;16(7):e0254511. doi: 10.1371/journal.pone.0254511 (PMC8277027; doi:10.1371/journal.pone.0254511)
Supplement: S3 Table — aNumber of respondents: items 1, 2 and 8, N = 144; items 3 and 5, N = 138; items 4 and 6, N = 117; item 7, N = 156; items 9 and 12, N = 134; item 10, N = 114; item 11, N = 123. bSignificant differences between before COVID-19 and outbreak (see Results for details) cSignificant differences between outbreak and de-escalation (see Results for details) Main contributors to significant differences are in bold typeset. A critical value of 0.00027 (i.e. Bonferroni correction for 12 survey items and 15 cells in each contingency table: 0.05/(12*15) = 0.00027) was selected; since adjusted residuals follow a normal distribution with mean = 0 and SD = 1, the critical value selected for adjusted residuals was 3.45. In conclusion, numbers in bold typeset point to those values whose adjusted residuals were greater than 3.45. (DOCX) [file pone.0254511.s006.docx]

|  | Before COVID-19^a^ | | | | | Outbreak (N=1109) | | | | | De-escalation (N=441) | | | | |
| --- | --- | --- | --- | --- | --- | --- | --- | --- | --- | --- | --- | --- | --- | --- | --- |
|  | 1 | 2 | 3 | 4 | 5 | 1 | 2 | 3 | 4 | 5 | 1 | 2 | 3 | 4 | 5 |
| 1^b^ | 11.8 | 16.7 | 18.8 | 19.4 | **33.3** | 6.2 | 35.4 | 18.8 | 33.9 | **5.7** | 7.3 | 36.1 | 21.5 | 31.7 | **3.4** |
| 2^c^ | 10.4 | 15.3 | 9.7 | 16.7 | 47.9 | 12.9 | 21.7 | 13.5 | 18.1 | **33.7** | 8.6 | 19.3 | 10.2 | 19.9 | 42 |
| 3^bc^ | **5.8** | 4.4 | 18.8 | 41.3 | 29.7 | 0.8 | 3 | 12 | 52.8 | 31.4 | 1.1 | 3 | 16.3 | 56.9 | 22.7 |
| 4^bc^ | **40.2** | 24.8 | 22.2 | **6.8** | 6 | 18.1 | 34.6 | 16.5 | **25** | 5.8 | 19.7 | 42.6 | 15.7 | 16.1 | 5.9 |
| 5 | 4.4 | 15.2 | 15.9 | 22.5 | 42 | 2.7 | 13.9 | 9 | 35.7 | 38.7 | 1.13 | 13.4 | 6.8 | 37.9 | 40.8 |
| 6^c^ | 8.5 | 9.4 | **32.5** | **15.4** | 34.2 | 5.7 | 12.3 | **17.1** | 37.9 | 27 | 8 | 14.7 | 24 | 37 | **16.3** |
| 7 | 78.2 | 12.2 | 0.6 | 3.2 | 5.8 | 75.5 | 16.6 | 2.2 | 2.3 | 3.4 | 73.2 | 17.7 | 1.4 | 2.7 | 5 |
| 8^c^ | 46.5 | 6.3 | 19.4 | 11.1 | 16.7 | **33.6** | 8.1 | 21.4 | 14.5 | **22.4** | **46.5** | 10.4 | **12.9** | 17.9 | **12.2** |
| 9 | 80.6 | 12.7 | 3.7 | 1.5 | 1.5 | 76.3 | 19.1 | 2.4 | 0.7 | 1.4 | 71.4 | 23.8 | 3.4 | 0.7 | 0.7 |
| 10^bc^ | **70.2** | 14 | 12.3 | 0.9 | 2.6 | **49.8** | 26.6 | 10.8 | 9.4 | 3.3 | 56.5 | 24.9 | 10.4 | 6.1 | 2.1 |
| 11^b^ | **52** | **28.5** | 12.2 | 3.2 | 4.1 | 18.6 | 46.9 | 20.1 | 11.4 | 3 | 17.9 | 53.3 | 17.7 | 7.9 | 3.2 |
| 12^b^ | **45.5** | **23.9** | 11.9 | 4.5 | 14.2 | 23.4 | 41 | 16.3 | 11.7 | 7.6 | 19.7 | **49** | 16.1 | 10.2 | 5 |
