# Supplementary material for: Polarization of beliefs as a consequence of the COVID-19 pandemic: The case of Spain
Source: PLoS One. 2021 Jul 13;16(7):e0254511. doi: 10.1371/journal.pone.0254511 (PMC8277027; doi:10.1371/journal.pone.0254511)
Supplement: S4 Table — Results are restricted to outbreak and de-escalation. Each model included item response (1 = strong agreement… 5 = strong disagreement) as dependent variable, COVID-19 sick acquaintance as predictor (= 1, yes; = 0, no), and wave, politics, sex, age, civil status and COVID-19 deceased relative as covariates. Number of observations = 1540. Note that positive values of z and OR greater than 1 indicate a stronger disagreement with the proposition. OR, odds ratio; SE, standard error. (DOCX) [file pone.0254511.s007.docx]

| Item 1 | Any failure can lead to a catastrophe | | | | | | |
| --- | --- | --- | --- | --- | --- | --- | --- |
|  | Model | LR χ^2^(14)=24.14, p=0.0441, pseudo-R^2^=0.0057 | | | | | |
|  |  |  | OR | SE | 95% CI | z | p |
|  |  | Sick acquaintance | 0.98 | 0.10 | 0.81,1.19 | -0.16 | 0.871 |
| Item 2 | **There is nothing beyond death** | | | | | | |
|  | Model | LR χ^2^(14)=347.12, p<0.0001, pseudo-R^2^=0.0741 | | | | | |
|  |  |  | OR | SE | 95% CI | z | p |
|  |  | Sick acquaintance | 1.35 | 0.13 | 1.11,1.64 | 3.06 | 0.002 |
| Item 3 | **The world is about to end** | | | | | | |
|  | Model | LR χ^2^(14)=27.31, p=0.0176, pseudo-R^2^=0.0080 | | | | | |
|  |  |  | OR | SE | 95% CI | z | p |
|  |  | Sick acquaintance | 1.17 | 0.12 | 0.95,1.43 | 1.52 | 0.129 |
| Item 4 | **Government authorities tend to be intrusive and controlling** | | | | | | |
|  | Model | LR χ^2^(14)=72.18, p<0.0001, pseudo-R^2^=0.0159 | | | | | |
|  |  |  | OR | SE | 95% CI | z | p |
|  |  | Sick acquaintance | 0.82 | 0.08 | 0.68,0.99 | -2.08 | 0.038 |
| Item 5 | **Scientific progress can help us overcome death and live forever** | | | | | | |
|  | Model | LR χ^2^(14)=126.82, p<0.0001, pseudo-R^2^=0.0316 | | | | | |
|  |  |  | OR | SE | 95% CI | z | p |
|  |  | Sick acquaintance | 1.38 | 0.14 | 1.13,1.67 | 3.20 | 0.001 |
| Item 6 | **Individual rights are more important than the needs of any group** | | | | | | |
|  | Model | LR χ^2^(14)=74.13, p<0.0001, pseudo-R^2^=0.0164 | | | | | |
|  |  |  | OR | SE | 95% CI | z | p |
|  |  | Sick acquaintance | 1.09 | 0.11 | 0.90,1.32 | 0.91 | 0.365 |
| Item 7 | **All human beings deserve respect** | | | | | | |
|  | Model | LR χ^2^(14)=57.89, p<0.0001, pseudo-R^2^=0.0232 | | | | | |
|  |  |  | OR | SE | 95% CI | z | p |
|  |  | Sick acquaintance | 0.74 | 0.09 | 0.57,0.94 | -2.42 | 0.016 |
| Item 8 | **God answers people’s prayers** | | | | | | |
|  | Model | LR χ^2^(14)=512.31, p<0.0001, pseudo-R^2^=0.1105 | | | | | |
|  |  |  | OR | SE | 95% CI | z | p |
|  |  | Sick acquaintance | 0.71 | 0.07 | 0.58,0.87 | -3.38 | 0.001 |
| Item 9 | **One should help those who are weak and cannot help themselves** | | | | | | |
|  | Model | LR χ^2^(14)=16.43, p=0.2878, pseudo-R^2^=0.0073 | | | | | |
|  |  |  | OR | SE | 95% CI | z | p |
|  |  | Sick acquaintance | 0.78 | 0.10 | 0.61,0.99 | -2.01 | 0.044 |
| Item 10 | **Being controlled or dominated by others is intolerable** | | | | | | |
|  | Model | LR χ^2^(14)=46.35, p<0.0001, pseudo-R^2^=0.0121 | | | | | |
|  |  |  | OR | SE | 95% CI | z | p |
|  |  | Sick acquaintance | 1.02 | 0.10 | 0.84,1.25 | 0.22 | 0.829 |
| Item 11 | **Most people generally have good intentions** | | | | | | |
|  | Model | LR χ^2^(14)=95.86, p<0.0001, pseudo-R^2^=0.0235 | | | | | |
|  |  |  | OR | SE | 95% CI | z | p |
|  |  | Sick acquaintance | 0.85 | 0.08 | 0.70,1.03 | -1.65 | 0.098 |
| Item 12 | **It is okay to use animals for medical research** | | | | | | |
|  | Model | LR χ^2^(14)=282.46, p<0.0001, pseudo-R^2^=0.0645 | | | | | |
|  |  |  | OR | SE | 95% CI | z | p |
|  |  | Sick acquaintance | 0.65 | 0.06 | 0.53,0.79 | -4.36 | <0.001 |
