# Supplementary material for: Polarization of beliefs as a consequence of the COVID-19 pandemic: The case of Spain
Source: PLoS One. 2021 Jul 13;16(7):e0254511. doi: 10.1371/journal.pone.0254511 (PMC8277027; doi:10.1371/journal.pone.0254511)
Supplement: S5 Table — Results are restricted to de-escalation (N = 441). Each model included item response (1 = strong agreement… 5 = strong disagreement) as dependent variable, COVID-19 deceased relative as predictor (= 1, yes; = 0, no), and politics, sex, age, civil status and COVID-19 sick acquaintance as covariates. Number of observations = 439. Note that positive values of z and OR greater than 1 indicate a stronger disagreement with the proposition. OR, odds ratio; SE, standard error. (DOCX) [file pone.0254511.s008.docx]

| Item 1 | Any failure can lead to a catastrophe | | | | | | |
| --- | --- | --- | --- | --- | --- | --- | --- |
|  | Model | LR χ^2^(13)=19.37, p=0.1120, pseudo-R^2^=0.0161 | | | | | |
|  |  |  | OR | SE | 95% CI | z | p |
|  |  | Deceased relative | 0.58 | 0.20 | 0.30,1.14 | -1.58 | 0.114 |
| Item 2 | **There is nothing beyond death** | | | | | | |
|  | Model | LR χ^2^(13)=135.19, p<0.0001, pseudo-R^2^=0.1065 | | | | | |
|  |  |  | OR | SE | 95% CI | z | p |
|  |  | Deceased relative | 1.17 | 0.43 | 0.57,2.40 | 0.44 | 0.662 |
| Item 3 | **The world is about to end** | | | | | | |
|  | Model | LR χ^2^(13)=13.79, p=0.3890, pseudo-R^2^=0.0141 | | | | | |
|  |  |  | OR | SE | 95% CI | z | p |
|  |  | Deceased relative | 0.60 | 0.22 | 0.29,1.23 | -1.39 | 0.165 |
| Item 4 | **Government authorities tend to be intrusive and controlling** | | | | | | |
|  | Model | LR χ^2^(13)=53.07, p<0.0001, pseudo-R^2^=0.0421 | | | | | |
|  |  |  | OR | SE | 95% CI | z | p |
|  |  | Deceased relative | 0.53 | 0.18 | 0.28,1.03 | -1.87 | 0.062 |
| Item 5 | **Scientific progress can help us overcome death and live forever** | | | | | | |
|  | Model | LR χ^2^(13)=59.39, p<0.0001, pseudo-R^2^=0.0547 | | | | | |
|  |  |  | OR | SE | 95% CI | z | p |
|  |  | Deceased relative | 0.91 | 0.32 | 0.45,1.82 | -0.28 | 0.780 |
| Item 6 | **Individual rights are more important than the needs of any group** | | | | | | |
|  | Model | LR χ^2^(13)=27.09, p=0.0121, pseudo-R^2^=0.0207 | | | | | |
|  |  |  | OR | SE | 95% CI | z | p |
|  |  | Deceased relative | 1.23 | 0.41 | 0.63,2.37 | 0.60 | 0.545 |
| Item 7 | **All human beings deserve respect** | | | | | | |
|  | Model | LR χ^2^(13)=23.67, p=0.0343, pseudo-R^2^=0.0320 | | | | | |
|  |  |  | OR | SE | 95% CI | z | p |
|  |  | Deceased relative | 1.04 | 0.09 | 0.44,2.46 | 0.09 | 0.924 |
| Item 8 | **God answers people’s prayers** | | | | | | |
|  | Model | LR χ^2^(13)=145.31, p<0.0001, pseudo-R^2^=0.1164 | | | | | |
|  |  |  | OR | SE | 95% CI | z | p |
|  |  | Deceased relative | 0.98 | 0.37 | 0.47,2.04 | -0.05 | 0.957 |
| Item 9 | **One should help those who are weak and cannot help themselves** | | | | | | |
|  | Model | LR χ^2^(13)=11.48, p=0.5708, pseudo-R^2^=0.0171 | | | | | |
|  |  |  | OR | SE | 95% CI | z | p |
|  |  | Deceased relative | 0.99 | 0.43 | 0.43,2.33 | -0.01 | 0.991 |
| Item 10 | **Being controlled or dominated by others is intolerable** | | | | | | |
|  | Model | LR χ^2^(13)=12.28, p=0.5051, pseudo-R^2^=0.0121 | | | | | |
|  |  |  | OR | SE | 95% CI | z | p |
|  |  | Deceased relative | 0.73 | 0.27 | 0.35,1.52 | -0.83 | 0.406 |
| Item 11 | **Most people generally have good intentions** | | | | | | |
|  | Model | LR χ^2^(13)=17.98, p=0.1582, pseudo-R^2^=0.0162 | | | | | |
|  |  |  | OR | SE | 95% CI | z | p |
|  |  | Deceased relative | 0.63 | 0.22 | 0.32,1.23 | -1.35 | 0.176 |
| Item 12 | **It is okay to use animals for medical research** | | | | | | |
|  | Model | LR χ^2^(13)=63.52, p<0.0001, pseudo-R^2^=0.0537 | | | | | |
|  |  |  | OR | SE | 95% CI | z | p |
|  |  | Deceased relative | 1.28 | 0.44 | 0.66,2.50 | 0.72 | 0.470 |
