# Supplementary material for: Polarization of beliefs as a consequence of the COVID-19 pandemic: The case of Spain
Source: PLoS One. 2021 Jul 13;16(7):e0254511. doi: 10.1371/journal.pone.0254511 (PMC8277027; doi:10.1371/journal.pone.0254511)
Supplement: S6 Table — Each model included a fixed-effects and a random-effects equation. Fixed effects: item response (1 = strong agreement… 5 = strong disagreement) as dependent variable, full interaction between wave and politics as predictors, and sex, older (= 0 if 18–40 yr, 1 = if >41 yr), single (= 0 if married/domestic partner; = 1 if single), COVID-19 sick acquaintance (1 = yes, 0 = no) and COVID-19 deceased relative (1 = yes, 0 = no) as covariates. Random effects: wave nested within subjects. Random effects were significant only for item 6 (χ2(1) = 7.95, p = 0.0024) Note that positive values of z and OR greater than 1 indicate a stronger disagreement with the proposition. OR, odds ratio; SE, standard error. (DOCX) [file pone.0254511.s009.docx]

| Item 4 | Government authorities tend to be intrusive and controlling | | | | | | |
| --- | --- | --- | --- | --- | --- | --- | --- |
|  | Model | Wald χ^2^(8)=22.51, p=0.0041 | | | | | |
|  |  |  | OR | SE | 95% CI | z | p |
|  | Wave |  |  |  |  |  |  |
|  | De-escalation (vs outbreak) | | 2.06 | 0.91 | 0.87,4.9 | 1.64 | 0.102 |
|  | Politics |  |  |  |  |  |  |
|  | Right (vs left voter) | | 3.74 | 1.53 | 1.68,8.33 | 3.22 | 0.001 |
|  | Wave × Politics | |  |  |  |  |  |
|  | De-escalation/Right | | 0.13 | 0.08 | 0.04,0.40 | -3.56 | <0.001 |
|  | Sex |  |  |  |  |  |  |
|  | Female | | 1.11 | 0.30 | 0.65,1.88 | 0.38 | 0.706 |
|  | Age |  |  |  |  |  |  |
|  | Older | | 1.07 | 0.36 | 0.56,2.06 | 0.21 | 0.837 |
|  | Civil status |  |  |  |  |  |  |
|  | Single | | 1.69 | 0.56 | 0.88,3.23 | 1.58 | 0.115 |
|  | COVID-19 sick |  |  |  |  |  |  |
|  | Yes | | 0.90 | 0.26 | 0.50,1.59 | -0.38 | 0.707 |
|  | COVID-19 deceased | |  |  |  |  |  |
|  | Yes | | 0.51 | 0.34 | 0.14,1.90 | -1.01 | 0.314 |
| Item 6 | **Individual rights are more important than the needs of any group** | | | | | | |
|  | Model | LR χ^2^(15)=75.31, p<0.0001, pseudo-R^2^=0.0167 | | | | | |
|  |  |  | OR | SE | 95% CI | z | p |
|  | Wave |  | 0.84 | 0.39 | 0.34,2.07 | -0.38 | 0.707 |
|  | De-escalation (vs outbreak) | |  |  |  |  |  |
|  | Politics |  |  |  |  |  |  |
|  | Right (vs left voter) | | 2.36 | 1.19 | 0.88,6.32 | 1.70 | 0.088 |
|  | Wave × Politics |  |  |  |  |  |  |
|  | De-escalation/Right | | 0.32 | 0.19 | 0.10,1.02 | -1.92 | 0.054 |
|  | Sex |  |  |  |  |  |  |
|  | Female | | 1.27 | 0.48 | 0.60,2.68 | 0.62 | 0.538 |
|  | Age |  |  |  |  |  |  |
|  | Older | | 0.64 | 0.31 | 0.25,1.65 | -0.92 | 0.358 |
|  | Civil status |  |  |  |  |  |  |
|  | Single | | 0.76 | 0.36 | 0.29,1.94 | -0.58 | 0.561 |
|  | COVID-19 sick |  |  |  |  |  |  |
|  | Yes | | 1.06 | 0.37 | 0.54,2.09 | 0.17 | 0.865 |
|  | COVID-19 deceased | |  |  |  |  |  |
|  | Yes | | 10.73 | 10.01 | 1.73,66.8 | 2.55 | 0.011 |
