# Supplementary figures and images for: Polarization of beliefs as a consequence of the COVID-19 pandemic: The case of Spain
Source: PLoS One. 2021 Jul 13;16(7):e0254511. doi: 10.1371/journal.pone.0254511 (PMC8277027; doi:10.1371/journal.pone.0254511)

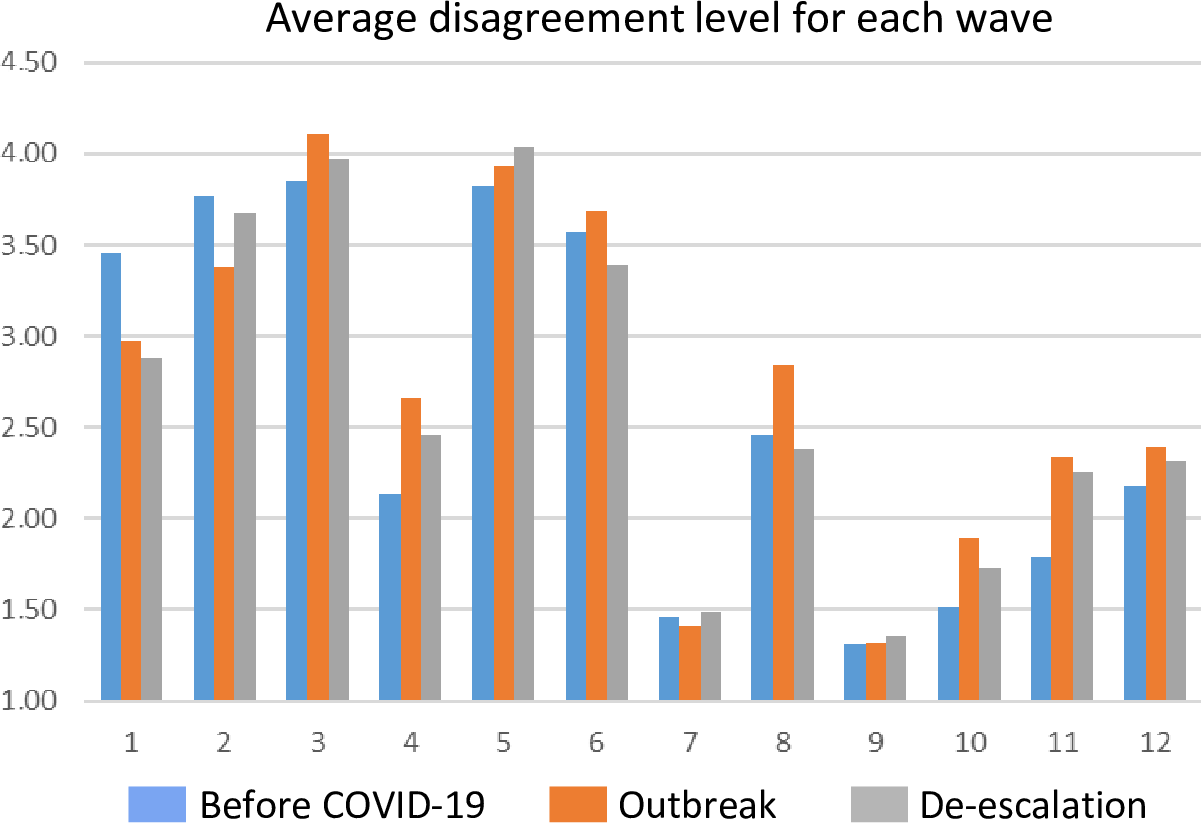

Supplement: S1 Fig — The value of each bar is calculated as the sum of the proportion of participants that responded each possible value (1 to 5) multiplied by that value. See S2 Table for a description of items. Note that higher values indicate a stronger disagreement with the proposition. (TIF) [file pone.0254511.s010.tif]

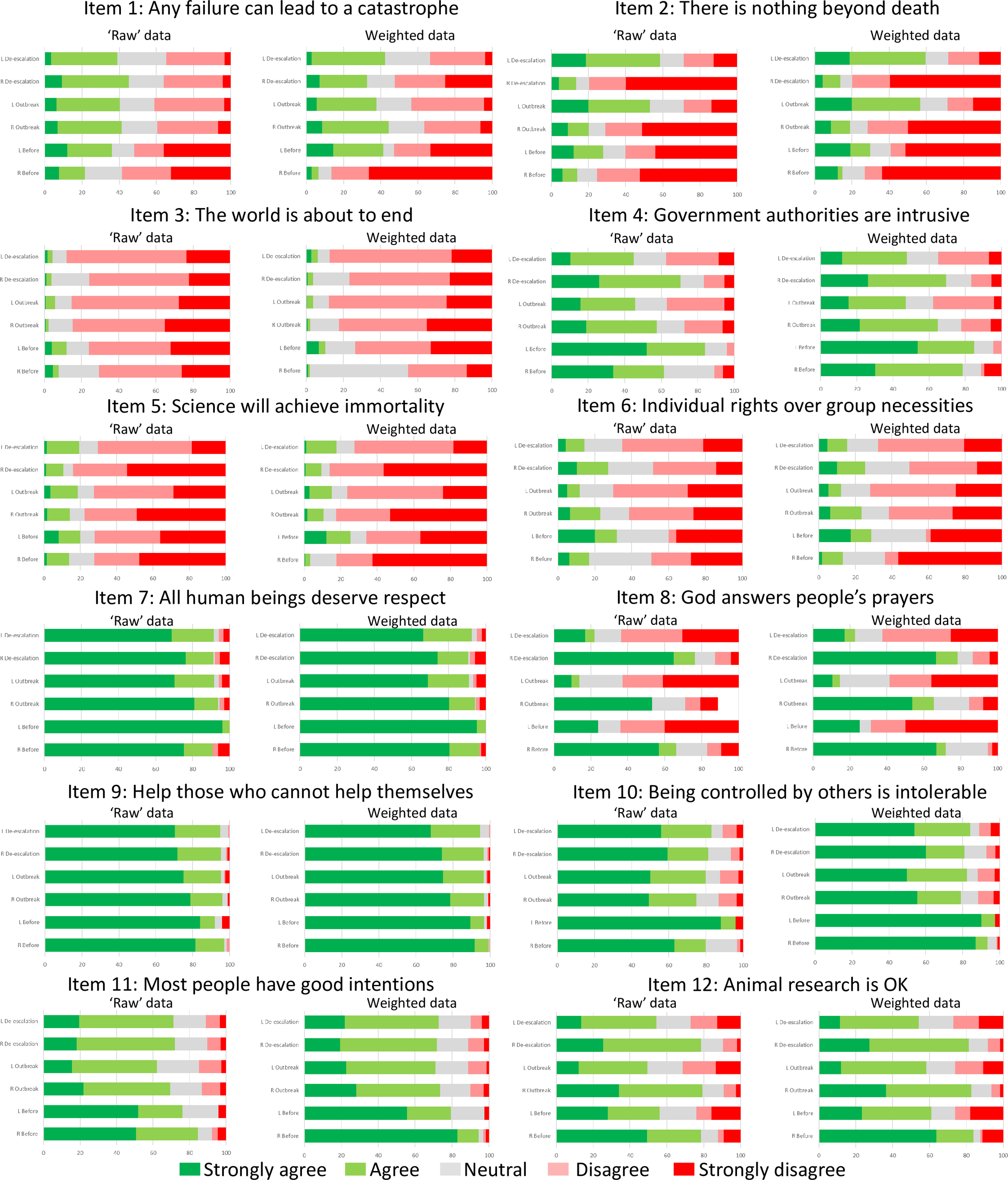

Supplement: S2 Fig — Stacked bars graphic showing the proportion of participants that responded to each disagreement level (from “strongly agree” to “strongly disagree”), stratified by wave and political preference (right-sided and left-sided voters), both with raw and weighted data (after iterative proportional fitting; see Materials and Methods). (TIF) [file pone.0254511.s011.tif]

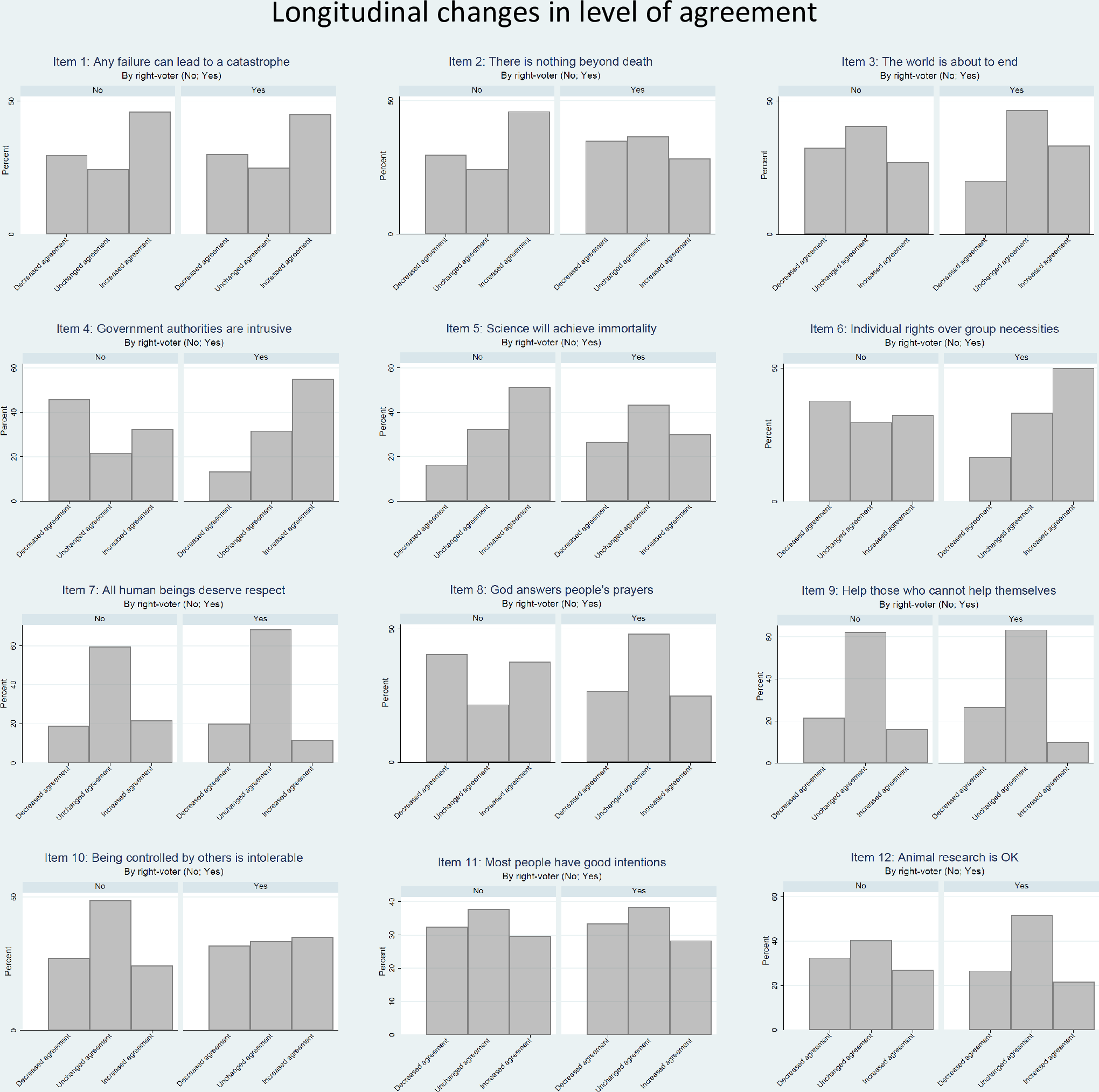

Supplement: S3 Fig — For each participant of the longitudinal dataset, responses to each item in the de-escalation were subtracted from those in the outbreak. Then, positive values were categorized as ‘increased agreement’, negative values as ‘decreased agreement’, and zeroes as ‘unchanged agreement’. Histograms shows the percentage of participants that increased, decreased or did not change their agreement between both waves, stratified by political preference (left, no right-sided voter; right, right-sided voter). (TIF) [file pone.0254511.s012.tif]
