# Supplementary material for: Polarization of beliefs as a consequence of the COVID-19 pandemic: The case of Spain
Source: PLoS One. 2021 Jul 13;16(7):e0254511. doi: 10.1371/journal.pone.0254511 (PMC8277027; doi:10.1371/journal.pone.0254511)
Supplement: Attachment — Submitted filename: Review to Impact of the COVID-19.docx [file pone.0254511.s013.docx]

This work presents the result from a sequential survey conducted in Spain across three temporal waves: one year before the COVID-19 pandemic, at its outbreak, and during de-escalation. Respondents were asked to declare their level of agreement with 12 propositions concerning transcendental, social or interpersonal subjects. The aim of the authors was to investigate the effect of the pandemic on the beliefs related to the propositions. They also investigated whether the effect was influenced by some features of the respondents: age, gender, civil status, political ideology, having an acquaintance affected of COVID-19, and a COVID-19 deceased relative.

The authors claim that:

- Despite the lockdown, respondents tolerated being controlled by authorities, and acknowledged the importance of group necessities over individual rights.
- De-escalation changed the above beliefs.
- Transcendental beliefs were strengthened.
- Left-sided voters did not see authorities as intrusive
- Transcendental beliefs prevailed among right-sided voters.
- The pandemic resulted in a polarization of belief system based on political ideology.

GENERAL EVALUATION

The study presented here is of the highest relevance. On the one hand, psychologists and sociologists will gain knowledge about the impact of events like the COVID-19 pandemic on the belief system of individuals. On the other hand, politicians and international organizations may use results of this kind to modulate their policies according to their expected impact on the population. I would really like to see studies like this one performed in other countries and when facing other traumatic events.

The paper is written in a clear way, with an accurate yet easy to follow English. The authors have made a big effort to dissect the results of the survey and extract from them relevant and potentially beneficial information regarding the effect of the pandemic on beliefs. I think that the claims made in the abstract are well addressed, and they are well supported by the results of the survey.

However, I am going to express two concerns about the main claims made by the authors. I will discuss other minor concerns and suggestions that, if addressed, I think will enrich this already superb work.

MAIN CONCERNS

- In the abstract, the authors claim that ‘left-sided voters did not see authorities as intrusive’. But in light of Fig 2, item 4, and Table S6, I think it is more accurate to say that the proportion of left-sided voters that agreed with the proposition ‘Government authorities are intrusive’ decreased dramatically, and the proportion that disagreed had the opposite trend (which is a striking result by itself). But still the level of agreement was slightly higher (or at least not any lower) than the level of disagreement.
- Similarly, in lines 360 and 361 of the Discussion, the authors claim that ‘during de-escalation, left-sided voters found tolerable being controlled by others’. But in lines 225-228 of the Results, as well as in Fig 2, item 10, what we see is that the proportion of left voters that agreed with the proposition ‘Being controlled by others is intolerable’ decreased dramatically (which, again, is a remarkable result). But still the level of agreement with the intolerability of being controlled by others is much higher.

In the two above cases, I think that slight rephrasing of the sentences might be enough. For example:

- ‘a remarkably higher proportion of left-sided voters did not see authorities as intrusive after the outbreak’.
- ‘during de-escalation, the proportion of left-sided voters that found tolerable being controlled by others remained overwhelmingly higher than before the pandemic’. And maybe add that for right-sided voters, despite a significant increase of disagreement during the outbreak, the levels were restored during the outbreak.

MINOR CONCERNS AND SUGGESTIONS

- Table S5 is a bit too crowded. I found a bit difficult to unpack the results from it. I would personally

find more accessible a figure composed of 12 panels (subplots). Each panel could include either five groups of three bars (one group per degree of agreement), or three groups of five bars (one group per wave). Although both options are equally informative, I would personally go for the latter.

- In addition, the evolution across waves of the average score of each proposition (sum of proportion of respondent times the level of agreement) could be shown.
- I have nothing to object to the analysis of the ‘transcendental’ questions (items 2 and 8), as the p values are the most reliable (or neutral) indicator of the existence of an effect. However, I think that the effect highlighted in the abstract is less remarkable than what visually stands out in Fig 1, and is pointed out in lines 331-333 of the discussion: during the outbreak the proportion of respondents that believed in an afterlife and in God answering people’s prayers decreased, while during de-escalation the tendency was reversed with a comparable strength. In the discussion it is reviewed how different groups may react differently to the pandemic, but I would suggest a discussion of the general effect I have just highlighted. To me, this could point to some interesting psychological effects, for example related to pessimism and nihilism during outbreak and lockdown, followed by optimism during de-escalation and drop in the number of deceased (many other interpretations are equally or more acceptable).
- Have the authors considered how question polarity (positive or negative wording of the sentence) influences the level of agreement? See for example in [Holleman, B., Kamoen, N., Krouwel, A., Pol, J. V. D., & Vreese, C. D. (2016). Positive vs. negative: The impact of question polarity in voting advice applications. *PloS one*, *11*(10), e0164184.], where It reads that the ‘choice to word questions positively (e.g., ‘The city council should allow cars into the city centre’) or negatively (‘The city council should ban cars from the city centre’) systematically affects the answers’.

For instance, if people is presented with the proposition ‘There is nothing beyond death’, they may be inclined to disagree to a higher extent than the agreement they would show when presented with the proposition ‘There is something after death’. Maybe the authors could comment on the criteria followed to formulate the propositions with a positive or a negative phrasing.

- Line 75: ‘At the time these lines are written’. For the sake of the reader’s curiosity, the (approximate) date could be stated.
- Line 211-213: ‘strongly agreed’ is used in the beginning of the sentence to describe the preference of those who either agreed or strongly agreed. A more accurate rewording could be ‘overwhelmingly agreed’.
- In lines 342-345 the attitude towards science is said to be partially critical because there is weaker ‘agreement with the capacity of science to achieve immortality’. But this seems more a question that evaluates faith in a very unlikely achievement, on which tiny proportion of researches may be working. I think that a more realistic question would have been about achieving perfect health until death, or extending life for centuries. Having said this, I am personally astonished by the high proportion of respondents that think that science will achieve immortality!
- In line 386, I am not sure that non-native English readers will understand the meaning of the word ‘cold’ in the context of the sentence.
- I have particularly enjoyed the paragraph starting in line 388. It is a clear exposition of how beliefs shape our attitudes and behaviours, and how understanding the ways in which traumatic global-scale events affect beliefs should be a priority of governments in the circumstance of adopting policies that involve public health and fundamental rights.
- In line 396, ‘Trump believers’ are mentioned. Maybe a very brief explanation of what this collective is would be adequate.
- Is the proportion of left and right-sided voters among the respondents similar to that of the total Spanish population? Could this have an influence on the results (for example Fig 1 or table S5)? An interesting test would be to add a newer version of Fig 1 and table S5, but correcting to the actual proportions in Spain, obtained from national surveys or the results of previous general elections.
- Regarding the title of papers, I personally find more powerful to capture in them the most striking result of the paper. In the case of the work discussed here, I could go for something like ‘Polarization on the belief system as a consequence of the COVID-19 pandemic: the case of Spain’.
- The longitudinal data are very interesting, and they well deserve a (supplementary) figure, similar to Figs 1 or 2.
- In table S5 the specific phrasing of the degrees of agreement is presented. Is not the use of ‘irrefutable’ a bit contradictory with the fact of not changing opinion? It remains me of the ‘Irresistible force paradox’ (What happens when an unstoppable force meets an immovable object?). Why the authors did not use a similar wording and phrasing than in degrees 2 and 4. For example ‘I agree, and I would not change my mind even if I were shown strong evidence’.
- Is the list of the 90 questions that comprised the original survey, and the answers to all of them, available somewhere else? If not, will they be available/published in the future? It would be very interesting for the reader of this paper to have access to them.
- In line 504, Statistical analyses, it reads ‘(…) selected dependent variables could significantly predict (…)’. I wonder whether the authors meant ‘independent variables’ instead.
- In line 521, Tables 3 and 4 are referenced regarding the ‘differences in predicted probabilities’. May be the authors actually referring to the ‘contrast’ results in Table S6?
