# Supplementary material for: Polarization of beliefs as a consequence of the COVID-19 pandemic: The case of Spain
Source: PLoS One. 2021 Jul 13;16(7):e0254511. doi: 10.1371/journal.pone.0254511 (PMC8277027; doi:10.1371/journal.pone.0254511)
Supplement: Attachment — Submitted filename: Response_to_Reviewers.docx [file pone.0254511.s015.docx]

# RESPONSE TO REVIEWERS (REVIEW #2)

Once again, we are extremely grateful to the Academic Editor and Reviewers for their helpful comments, which we fully address in the revised version of our manuscript as follows.

ACADEMIC EDITOR:

Reviewer 1, however is questioning the analysis represented and interpreted in Table 2.  The footnote and text say that the analyses include an interaction term for wave x political preference.  If that is the case, then the main effects of wave should not be interpreted.  Reviewer 2 suggests running the same analyses and presenting them both with and without the interaction, and I agree.  Reviewer 2 has some additional minor suggestions -- please attend to these edits.

**Response**: We have included all suggestions raised by Reviewer 2 (see below). We are extremely grateful for having detected this misinterpretation of our analyses. Please note that results do not substantially change (Table 2; changes in pp. 12-13 (Methods), and pp. 14, 15 and 17 in Results; page numbers refer to text with track changes). We have also corrected the analyses pertaining participants with a COVID-19 sick acquaintance, since they had the same problem (pp. 20-22).

In addition to the reviewers' comments, I would add that lines 302-309 on p. 13 constitute results, and therefore belong in the results section.

**Response**: We have removed these lines from the Methods (p. 13).

Also, there is a typo in the note to Table 3, line 397 - "that" should be "than".

**Response**: Thanks. This typo has been corrected.

In addition, we have referenced two supplementary materials that were missing in the previous version of the manuscript (that is, they were correctly uploaded, but they were not in the list of Supplementary Information at the end of the manuscript): S1 Datasets and S1 Statistical reports (p. 38). We have also corrected some typos.

Reviewer #1: Overall

- This revision is a significant improvement on the original submission. I appreciate the inclusion of a research question and hypothesis in the introduction. The research methods are also described clearly and I arrived at the results section with a much better idea of the data and plan for analysis. The figures were also much easier to interpret. However, there remains one major issue and a few points of clarification before this manuscript can be accepted. Note: all page numbers refer to document without track changes.

Major issue

- The first models presented in Fig 1, Table 2, and S2 Table talk about the effect of wave on beliefs. However, based on the sentence that starts on p14 line 342 and the caption to Table 2, it sounds like these models also include the interaction of political preference and wave. If correct, the inclusion of an interaction term radically changes the interpretation of any “main effect” of wave on belief. As currently specified, I think the main effects for wave only hold when politics = 0 (“right voter”) and thus do not convey the general effect of the pandemic across all voters. The easiest solution would be to simply rerun the models without the interaction effect (reproducing Fig 1, Table 2, and S2 Table) and then include the interaction effect for the subsequent discussion of results that begins on p17 “Effect of political preferences on pandemic-related belief changes.”

**Response**: We are extremely grateful for noticing this. Indeed, “main effects” are not interpretable when there is an interaction term involving one or several of the variables included in the interaction. As the Reviewer suggests, the “main effects” we were reporting only referred to politics=0, that is, to right-sided voters. We followed the solution suggested by the Reviewer, and repeated the analyses without the interaction term for that section of the Results. Thus, numbers in Table 2 have been completely changed (we have not used track changes for that). Please note that results have not changed substantially. In fact, just a few changes were necessary in the text (changes in pp. 12-13 (Methods), and pp. 14, 15 and 17 in Results; page numbers refer to text with track changes). We have also corrected the analyses pertaining participants with a COVID-19 sick acquaintance, since they had the same problem (pp. 20-22).

Minor notes

- p4 line 93: extra period.

**Response**: This has been corrected

- p10 line 243: avoid using “degree” when describing agreement or disagreement. “Level” would work better here. Same issue on p14 line 339.
**Response**: Thanks for this suggestion. We have substituted all instances of “degree” (in this context) for “level”.

- p11: operationalization of belief vs opinion seems to depart from conventional uses of the word “belief” but will trust the cited source here.
**Response**: Thank you.

- I still need help reading Table 3 and Table 4. Are the “contrast” values the changes in predicted probability? I had trouble moving between Table 4 and the main text on p25 line 561-562: where does the 40% to 78.3% come from? An extra sentence or two of clarification are needed here.
**Response**: We understand the confusion. As expressed in the footnotes of Tables 3 and 4, “contrast” refers to the change in the predicted probability to respond 1 (columns on the left) or 5 (columns on the right). We decided to display only these results for the sake of clarity. We have highlighted this part of the footnote by adding an asterisk on “Contrast” in both tables. However, the percentages mentioned by the Reviewer do not come from the changes in the predicted probabilities: those are ‘actual’ (and not predicted) percentages of the longitudinal data, which are not directly shown in any table or figure (although they can be easily calculated from the datasets. The Reviewer’s confusion is absolutely justified, and this comment is a good opportunity to clarify our results. First of all, we now include the Stata log with these description (longitudinal dataset: proportion of each disagreement level by wave by politics, for each item). This is included in S1 Statistical Reports. Second, we clearly explain where those percentages are coming from: “Also, the percentage of respondents that expressed each disagreement level for every item is shown in S1 Statistical reports, sorted by political ideology (right-sided voter, yes/no) and wave (outbreak/de-escalation). With regards to these data,…” (p. 26). Finally, changes in predicted probabilities are more clearly explained: “This effect was also found in the predicted probabilities of the multilevel model shown in Table 4: for right-sided voters, there was a 19.3% increased probability of strongly agreeing with authorities being intrusive in the de-escalation with respect to the outbreak; however, for non-right-sided voters, there was a 12.3% decreased probability of strongly agreeing in the de-escalation than in the outbreak” (pp. 26-27).

- p26 line 601: if this is the “first report” on belief change and COVID-19, what about citation #24?

**Response**: This is correct. This sentence has been rewritten: “The main novelty of this research is to assess belief changes due to the COVID-19 pandemic across several domains, which is especially valuable for having been carried out in one of the first and most affected countries.” (p. 28).

- p29 line 676-678: study should be properly cited in reference section.

Done (p. 31).

Reviewer #2: The authors have done an awesome work on addressing the concerns expressed by the reviewers. I think that the paper is ready to be published as it stands now. It will be an extremely valuable contribution and will help understand the psychological and sociological foundations of beliefs.

P.S. I think there is a typo in line 671 of the Discussion (version without track changes active). It reads ‘other’ when it should read ‘others’.

**Response**: Thank you for noticing this. We have corrected this typo.
